# ASO Author Reflections: Advancements in Esophageal Cancer Surgery—A Swedish Perspective

**DOI:** 10.1245/s10434-025-17128-9

**Published:** 2025-03-14

**Authors:** Ellinor Jenny Gunilla Lundberg, Fredrik Mattsson, Eivind Gottlieb-Vedi, Jesper Lagergren

**Affiliations:** 1 Molecular Medicine and Surgery, Karolinska Institutet, Stockholm, Sweden; 2 King’s College, London, UK

## Past

Esophageal cancer has historically been associated with very poor survival outcomes. Treatment has developed over the last decades with surgical refinements, multimodal treatment approaches, and novel neoadjuvant or perioperative therapies, as well as strategies for better patient selection and centralization of care. This has led to improved survival rates, but the survival remains suboptimal.^1,2^ There is a lack of population-based studies that have evaluated the recent long-term survival trends and examined potential contributing factors. Our study aimed to fill this knowledge gap by analyzing nationwide data on patients undergoing esophagectomy in Sweden between 2000 and 2020 with follow-up until 2024.^3^

## Present

Our findings demonstrate a significant improvement in 5-year survival during the last years following esophagectomy for esophageal cancer in Sweden. The hazard ratio for all-cause 5-year mortality continuously declined over time, with a 43% lower risk in 2015–2020 compared with 2000–2004.^3^ This improvement occurred despite increasing overall resection rates and more frequent surgery on patients of older age, with more comorbidity and with more advanced tumor stage during later calendar periods. Instead, factors such as advancements in preoperative imaging, perioperative treatment, introduction of immunotherapy, surgical standardization, and centralization to high-volume centers may have contributed.

## Future

Despite these promising trends, further research is needed to optimize patient selection and refine surgical approaches. While cancer surgery is increasingly performed on older and more comorbid patients with encouraging outcomes,^4^ the risk–benefit balance of surgery in high-risk individuals remains a key area for investigation.
